# Synthesis of a Novel D-Glucose-Conjugated 15-Crown-5 Ether with a Spiro Ketal Structure

**DOI:** 10.3390/molecules13081840

**Published:** 2008-08-22

**Authors:** Takashi Yamanoi, Yoshiki Oda, Hitomi Muraishi, Sho Matsuda

**Affiliations:** The Noguchi Institute, 1-8-1 Kaga, Itabashi-ku, Tokyo 173-0003, Japan; E-mails: odayoshiki@noguchi.or.jp (Yoshiki Oda); hitommy-heart@bridge.ocn.ne.jp (Hitomi Muraishi); s.matsuda@noguchi.or.jp (Sho Matsuda)

**Keywords:** Crown ether, Spiroketal, 1-*C*-Vinylated glucose, Glycosylation

## Abstract

This paper describes a synthetic approach to a novel D-glucose-conjugated 15-crown-5 ether having a spiroketal structure starting from a 1-*C*-vinylated glucose derivative. The approach consists of the glycosylation of the vinylated glucose derivative to give an ethyleneoxy spacer derivative using bismuth(III) triflate, the conversion of the 1-*C*-vinyl group of the glucoside produced into a carboxylic acid group, and the intramolecular condensation between the carboxyl group and the terminal hydroxyl group in the ethyleneoxy spacer. A D-glucose-conjugated 15-crown-5 ether having a unique spiroketal structure was thus successfully synthesized.

## Introduction

Crown ether molecules with saccharide moieties are interesting as chiral phase-transfer catalysts [1,2]. An enzymatic approach for synthesizing these types of crown ethers provides the cyclofructan family (cycloβ(2→1)-D-fructooligosaccharides) via the digestion of inulin. The cyclofructan contains a structurally interesting crown ether framework in its central core [3,4]. It is noteworthy that this is the first example of saccharide-based crown ethers which have spiroketal structures. Many saccharide-based crown ether molecules have also been synthesized by chemical procedures [5,6,7]. As these chemical methods bind the original hydroxyl groups of the saccharide with an ethyleneoxy spacer, they cannot produce however crown ether compounds having spiroketal structures.

Sugar derivatives (1-*C*-vinylated sugars) having a vinyl group at the anomeric center, which are readily prepared by the addition of organometallic reagents, such as vinylMgX, to a suitably protected sugar lactone, are a synthetically useful tool in carbohydrate chemistry [8,9,10,11]. Our recent studies have shown that these 1-*C*-vinylated sugar derivatives were good precursors for preparing some fuctionalized *exo*-glycal derivatives [12] and naturally occurring anhydroketopyranoses [13]. For the purpose of further exploring the utility of the 1-*C*-vinylated sugars, we investigated the synthesis of a novel crown ether molecule from a 1-*C*-vinylated D-glucose derivative **1**. The D-glucose-conjugated 15-crown-5 ether **2** that we designed is a dicyclic compound with a unique spiroketal structure derived from the structural characteristic of **1**, *i.e.,* its spiro carbon atom corresponds to the anomeric carbon atom. This paper describes our synthetic approach to a novel 15-crown-5 ether **2** having a spiroketal structure from a 1-*C*-vinylated glucose derivative (**1**).

## Results and Discussion

**Scheme 1:**
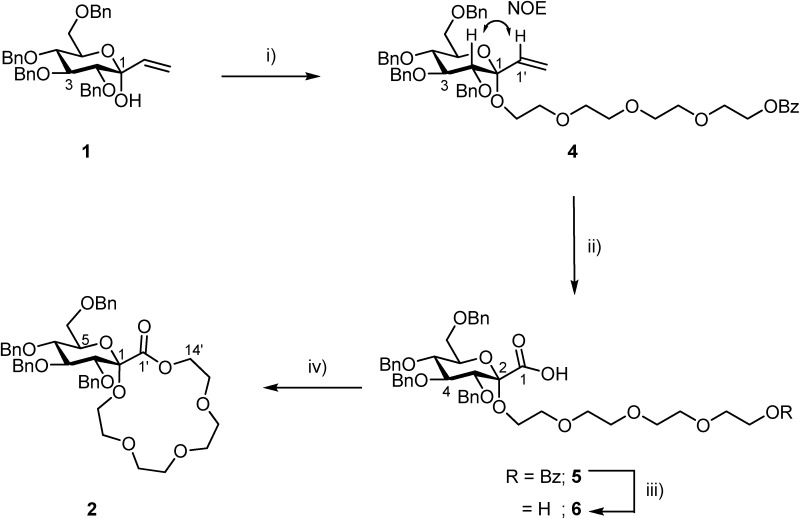
Synthetic approach to **2**.

The synthetic approach to compound **2** from **1** is shown in Scheme 1. It consists of the following reaction steps: 1) introduction of the ethyleneoxy spacer, tetraethyleneglycol monobenzoate (**3**) onto the vinylated D-glucopyranose derivative **1** by the glycosylation reaction; 2) conversion of the vinyl group at the anomeric center of **4** to a carboxyl group, and 3), intramolecular condensation between the carboxyl group and the terminal hydroxyl group in the ethyleneoxy spacer to produce the desired **2**.

The glycosylation of **1** to **3** (1.3 equiv.) using bismuth(III) triflate (Bi(OTf)_3_) (0.05 equiv.) in the presence of anhydrous CaSO_4_ in dichloromethane at 0 ^o^C for 24 h afforded the desired glucoside **4**[14], which was purified by preparative TLC (ethyl acetate/hexane = 1/2) in 81% yield. The glycosylation proceeded with an α-stereoselectivity. The high α-stereoselectivity of the glycosylation using **1** was in agreement with our previously reported observation [15]. The α-anomeric configuration of **4** was determined by the NOE interaction between the H-2 and the H-1’.

The ozone oxidation of **4** in dichloromethane at -78 ^o^C for 5 h and treatment with triphenylphosphine (3.4 equiv.) at room temperature for 19 h gave the crude aldehyde product. The subsequent oxidation using NaClO_2_ (12 equiv.)-NaH_2_PO_4_ (3 equiv.) in *t*-butyl alcohol-H_2_O (4/1) produced the carboxylic acid derivative **5**, which was purified by preparative TLC (CHCl_3_/MeOH = 5/1) in 85% yield.

Deprotection of the benzoyl group of **5** was performed using 0.5 M NaOH/THF to afford **6** in 83% yield. The cyclization of **6** using (benzotriazol-1-yloxy)tripyrrolidinophosphonium hexafluoro-phosphate (PyBOP) (2.5 equiv.) and DIEA (1.8 equiv.) in dichloromethane for 24 h afforded the desired **2**, which was purified by preparative TLC (CHCl_3_/MeOH = 20/1) in 84% yield.

In conclusion, we have demonstrated the synthesis of a novel 15-crown-5 ether **2** having a spiro ketal structure from a 1-*C*-vinylated D-glucose derivative. This compound **2** is expected to function as a chiral phase-transfer catalyst.

*Reagents and conditions*: i) Bi(OTf)_3_ (0.05 equiv.), Bz(OCH_2_CH_2_)_4_OH **3** (1.3 equiv.), CH_2_Cl_2_, 0 °C, 24 h, 81%; ii) (a) O_3_, -78 °C, 5 h, CH_2_Cl_2_, then Ph_3_P (3.4 equiv.), rt, 19 h. (b) Me_2_C=CHMe (4.5 equiv.), NaClO_2_ (12 equiv.), NaH_2_PO_4_ (3 equiv.), *t*-BuOH-H_2_O, rt, 24 h, 85%; iii) 0.5 M NaOH (13 equiv.), THF, rt, 3 h, 83%; iv) PyBOP (2.5 equiv.), DIEA (1.8 equiv.), CH_2_Cl_2_, 24 h, 84%.

## Experimental

### General

^1^H-NMR (600 MHz) and ^13^C-NMR (150 MHz) spectra were recorded using a JEOL ECA-600 spectrometer in CDCl_3_ with TMS as the internal standard. The optical rotations were recorded by a JASCO DIP-360 digital polarimeter. The HRMS were obtained using a Mariner spectrometer (PerSeptive Biosystems Inc.). Preparative TLC was performed using Merck silica gel 60GF254. Column chromatography was conducted using silica gel 60 N (40~50 μm, Kanto Chemical Co., Inc.). Bi(OTf)_3_ was purchased from Sigma-Aldrich. All anhydrous solvents were purified according to standard methods.

*11-Benzoyloxy-3,6,9-trioxaundecyl 2,3,4,6-tetra-O-benzyl-1-C-vinyl-α-D-glucopyranoside* (**4**): To a stirred solution of Bi(OTf)_3_ (15 mg, 0.023 mmol) in CH_2_Cl_2_ (3.5 mL) were added tetraethyleneglycol monobenzoate (**3**) (165 mg, 0.55 mmol) and 2,3,4,6-tetra-*O*-benzyl-1-*C*-vinyl-α-D-glucopyranose (**1**) (257 mg, 0.42 mmol) in the presence of anhydrous CaSO_4_ (280 mg) under an Ar atmosphere. The resulting mixture was stirred at 0 ^o^C for 24 h. The reaction was then quenched by the addition of a sat. NaHCO_3_ solution (5 mL). The reaction mixture was extracted with CH_2_Cl_2_ (three times), and the organic layer was washed with water and a sat. NaCl solution. After the organic layer was dried over Na_2_SO_4_, the solvent was evaporated under reduced pressure. The crude product was purified by preparative silica gel TLC (ethyl acetate/hexane = 1/2) to give **4** (311 mg, 81% yield) as a colorless oil. [α]_D_^25^ = +3^o^ (*c* 4.7, CHCl_3_); ^1^H-NMR: δ 3.34 (d, 1H, *J* = 9.6 Hz, H-2), 3.48-3.70 (m, 14H, H-4, H_a_-6, C*H*_2_CH_2_), 3.76-3.79 (m, 3H, H_b_-6, C*H*_2_CH_2_), 3.85 (m, 1H, H-5), 4.10 (t, 1H, *J* = 9.7 Hz, H-3), 4.43-4.91 (m, 8H, C*H_2_*Ph), 5.27 (dd, 1H, *J* = 2.0 Hz, *J* = 11.0 Hz, CH=C*H_a_*H_b_), 5.54 (dd, 1H, *J* = 2.1 Hz, *J* = 17.9 Hz, CH=CH_a_*H_b_*), 5.99 (m, 1H, C*H*=CH_2_), 7.19-7.54, 8.04-8.05 (m, 25H, Ph); ^13^C-NMR: δ 61.1 (*C*H_2_CH_2_), 64.1 (*C*H_2_CH_2_), 68.8 (C-6), 69.2 (*C*H_2_CH_2_), 70.0 (*C*H_2_CH_2_), 70.6 (*C*H_2_CH_2_), 70.64 (*C*H_2_CH_2_), 70.7 (*C*H_2_CH_2_), 71.5 (C-5), 73.4 (*C*H_2_Ph), 75.0 (*C*H_2_Ph), 75.5 (*C*H_2_Ph), 75.8 (*C*H_2_Ph), 78.5 (C-4), 83.0 (C-3), 84.3 (C-2), 99.5 (C-1), 118.8 (CH=*C*H_2_), 127.5-133.0 (Ph), 135.3 (*C*H=CH_2_), 138.1-138.4 (Ph), 166.5 (C=O); HRMS (ESI) *m/z* calcd for C_51_H_58_NaO_11_ 869.3871 [M +Na]^+^, found 869.3865.

*(11-Benzoyloxy-3,6,9-trioxaundecyl 3,4,5,7-tetra-O-benzyl-α-D-gluco-hept-2-ulopyranosid)onic acid* (**5**): Ozone was bubbled through a stirred solution of **4** (224 mg, 0.26 mmol) in CH_2_Cl_2_ (15 mL) at -78 ^o^C for 5 h. After triphenylphosphine (230 mg, 0.88 mmol) was added at -78^ o^C and the reaction temperature was raised to room temperature, the reaction mixture was stirred for 19 h. The solvent was then evaporated under reduced pressure. To a solution of the crude product in *t*-butyl alcohol (4 mL)-H_2_O (1 mL) were added NaClO_2_ (277 mg, 3.1 mmol), NaH_2_PO_4_ (124 mg, 0.8 mmol) and 2-methyl-2-butene (123 μL, 1.2 mmol). After the reaction mixture was stirred for 24 h, the reaction was quenched by adding 2 M HCl (1 mL) and water (5 mL). The reaction mixture was then extracted with CH_2_Cl_2_ (three times), and the combined organic solvent was dried over anhydrous Na_2_SO_4_. The organic solvent was filtered and evaporated under reduced pressure. The crude product was purified by preparative silica gel TLC (CHCl_3_/MeOH = 5/1) to afford **5** (194 mg, 85% yield) as a colorless oil. [α]_D_^25^ = +21^o^ (*c* 3.9, CHCl_3_); ^1^H-NMR: δ 3.54-4.08 (m, 20H, H-3, H-4, H-5, H-6, H-7, C*H*_2_CH_2_), 4.41-4.49 (m, 2H, C*H*_2_CH_2_), 4.51-5.28 (m, 8H, C*H_2_*Ph), 7.03-7.53 (m, 23H, Ph), 8.03-7.53 (d, 2H, *J* = 6.8 Hz, Ph); ^13^C-NMR: δ 64.0 (*C*H_2_CH_2_), 69.1 (C-7), 69.9 (*C*H_2_CH_2_), 70.2 (*C*H_2_CH_2_), 70.3 (*C*H_2_CH_2_), 70.4 (*C*H_2_CH_2_), 70.5 (*C*H_2_CH_2_), 70.6 (*C*H_2_CH_2_), 72.6 (*C*H_2_CH_2_), 75.19 (*C*H_2_Ph), 75.20 (*C*H_2_Ph), 75.4 (*C*H_2_Ph), 75.5 (*C*H_2_Ph), 77.6 (C-6), 80.9 (C-5), 82.7 (C-3, C-4), 99.3 (C-2), 126.0-139.2 (Ph), 166.5 (*C*(O)Ph), 177.7 (C-1); HRMS (ESI) *m/z* calcd for C_50_H_56_NaO_13_ 887.3613 [M +Na]^+^, found 887.3653.

*(11-Hydroxy-3,6,9-trioxaundecyl 3,4,5,7-tetra-O-benzyl-α-D-gluco-hept-2-ulopyranosid)onic acid* (**6**): A 0.5 M NaOH solution (4 mL, 2 mmol) was added to a solution of **5** (142 mg, 0.16 mmol) in THF (4 mL). After the reaction mixture was stirred for 3 h at room temperature, the reaction was quenched by adding 2 M HCl (1 mL) and water (5 mL). After the reaction mixture was extracted with CH_2_Cl_2_ (three times), the combined organic solvent was dried over anhydrous Na_2_SO_4_. The organic solvent was filtered and evaporated under reduced pressure. The crude product was purified by preparative silica gel TLC (CHCl_3_/MeOH = 5/1) to afford **6** (103 mg, 83% yield) as a colorless oil. [α]_D_^25^ = +25^o^ (*c* 1.8, CHCl_3_); ^1^H-NMR: δ 3.37-4.00 (m, 22H, H-3, H-4, H-5, H-6, H-7, C*H*_2_CH_2_), 4.44-4.84 (m, 8H, C*H_2_*Ph), 6.94-7.43 (m, 20H, Ph); ^13^C-NMR: δ 60.4 (*C*H_2_CH_2_), 62.6 (*C*H_2_CH_2_), 68.5-70.4 (*C*H_2_CH_2_, C-7), 72.4 (*C*H_2_Ph), 73.4 (*C*H_2_Ph), 74.9 (*C*H_2_Ph), 75.3 (*C*H_2_Ph), 78.1 (C-6), 82.4 (C-5), 82.9 (C-3, C-4), 99.7 (C-2), 127.3-128.4, 137.8-138.9 (Ph), 172.3 (C-1); HRMS (ESI) *m/z* calcd for C_43_H_52_NaO_12_ 783.3351 [M +Na]^+^, found 783.3396.

*(1R)-2,3,4,6-Tetra-O-benzylspiro[1,5-anhydro-D-glucitol-1,2’-[3,6,9,12]tetraoxatetradecan]-14’-olide* (**2**): To a solution of **6** (20 mg, 0.027 mmol) in CH_2_Cl_2_ (3 mL) were added 4-dimethylaminopyridine (5.9 mg, 0.048 mmol) and PyBOP (35 mg, 0.067 mmol). After the reaction mixture was stirred for 24 h. The reaction was then quenched by the addition of a sat. citric acid solution (5 mL). The reaction mixture was extracted with EtOAc and the organic layer was washed with water and a sat. NaCl solution. After the organic layer was dried over Na_2_SO_4_, the solvent was evaporated under reduced pressure. The crude product was purified by preparative silica gel TLC (CHCl_3_/MeOH = 20/1) to give **2** (17 mg, 84% yield) as a colorless oil. [α]_D_^25^ = +11^o^ (*c* 0.15, CHCl_3_); ^1^H-NMR: δ 3.48-3.71 (m, 15H, H-4, H-5, H-6, C*H*_2_CH_2_), 3.73-3.75 (m, 1H, C*H*_2_CH_2_), 3.81 (d, 1H, *J* = 9.6 Hz, H-2), 3.87-3.92 (m, 1H, C*H*_2_CH_2_), 3.94-3.98 (m, 1H, C*H*_2_CH_2_), 4.00-4.05 (m, 1H, C*H*_a_H_b_CH_2_), 4.06-4.07 (m, 1H, H-3), 4.31-4.34 (m, 1H, CH_a_*H*_b_CH_2_), 4.54-4.65 (m, 4H, C*H_2_*Ph), 4.79-4.89 (m, 4H, C*H_2_*Ph), 7.16-7.35 (m, 20H, Ph); ^13^C-NMR: δ 63.6 (*C*H_2_CH_2_), 65.2 (*C*H_2_CH_2_), 68.3 (*C*H_2_CH_2_), 68.4 (C-6), 69.6 (*C*H_2_CH_2_), 70.1 (*C*H_2_CH_2_), 70.4 (*C*H_2_CH_2_), 70.9 (*C*H_2_CH_2_), 71.2 (*C*H_2_CH_2_), 73.41 (C-5), 73.44 (*C*H_2_Ph), 75.1 (*C*H_2_Ph), 75.2 (*C*H_2_Ph), 75.6 (*C*H_2_Ph), 78.0 (C-4), 82.2 (C-2), 82.7 (C-3), 99.7 (C-1), 127.5-128.4 (Ph), 137.9-138.5 (Ph), 173.5 (C-1’); HRMS (ESI) *m/z* calcd for C_43_H_50_NaO_11_ 765.3245 [M +Na]^+^, found 765.3247.

## References

[1] Mako A., Szollosy A., Keglevich G., Menyhard D.K., Bako P., Toke L. (2008). Synthesis of methyl-α-D-glucopyranoside-based azacrown ethers and their application in enantioselective reactions. Monatsh. Chem..

[2] Itoh T., Shirakami S. (2001). Synthesis of chiral crown ethers derived from α-D-glucose and their catalytic properties on the asymmetric michael addition. Heterocycles.

[3] Takai Y., Okumura Y., Tanaka T., Sawada M., Takahashi S., Shiro M., Kawamura M., Uchiyama T. (1994). Binding characteristics of a new host family of cyclic oligosaccharides from inulin: Permethylated cycloinulohexaose and cycloinulohepaose. J. Org. Chem..

[4] Louis F., Garcia-Moreno M.I., Balbuena P., Mellet C.O., Garcia-Fernandez J.M. (2008). Stereoselective synthesis of nonsymmetrical difructose dianhydrides from xylylene-tethered D-fructose precursors. Tetrahedron.

[5] Mani N.S., Kanakamma P.P. (1994). Synthesis of novel chiral macrocycles: Crown ethers derived from D-glucose. Tetrahedron Lett..

[6] Faltin F., Fehring V., Miethchen R. (2002). Chiral crown ethers based on galactopyranosides. Synthesis.

[7] Dumont-Hornebeck B., Joly J.P., Coulon J., Chapleur Y. (1999). Synthesis of ethoxy-linked pseudo-disaccharides incorporating a crown ether macrocycles and lectin recognition. Carbohydr. Res..

[8] Tomooka K., Nakamura Y., Nakai T. (1995). [2, 3]-Wittig rearrangement using glucose as a chiral auxiliary: asymmetric transmission from the anomeric center. Synlett..

[9] Lay L., Meldal M., Nicotra F., Panza L., Russo G. (1997). Stereoselective synthesis of the *C*-analogue of β-D-glucopyranosyl serine. Chem. Commun..

[10] van Hooft P.A.V., Oualid F.E., Overkleeft H.S., van der Marel G.A., van Boom J.H., Leeuwenburgh M.A. (2004). Synthesis and elaboration of functionalised carbohydrate-derived spiroketals. Org. Biomol. Chem..

[11] Li X.L., Ohtake H., Takahashi H., Ikegami S. (2001). A facile synthesis of 1′-*C*-alkyl-α-disaccharides from 1-*C*-alkyl-hexopyranoses and methyl 1-*C*-methyl-hexopyranosides. Tetrahedron.

[12] Yamanoi T., Nara Y., Matsuda S., Oda Y., Yoshida A., Katsuraya K., Watanabe M. (2007). Synthetic approach to *exo*-glycals from 1-*C*-vinyl-d-glycopyranose derivatives via an S_N_1′-substitution mechanism. Synlett..

[13] Matsuda S., Matsumura K., Watanabe M., Yamanoi T. (2007). Synthesis of a partially benzylated derivative of the anhydro-D-altro-heptulose found in *Coriaria japonica* A. Tetrahedron Lett..

[14] Yamanoi T., Inoue R., Matsuda S., Katsuraya K., Hamasaki K. (2006). Synthesis of trehalose mimics by bismuth(III) triflate or bis(trifluoromethane)sulfonimide-catalyzed 1-*C*-methyl-D-hexopyranosylation. Tetrahedron Asymmetry.

[15] Yamanoi T., Oda Y., Matsuda S., Yamazaki I., Matsumura K., Katsuraya K., Watanabe M., Inazu T. (2006). Synthesis of 1-*C*-alkyl-α-D-glucopyranosides by Lewis acid- or Brønsted acid-catalyzed O-glycosidation. Tetrahedron.

